# Under-five mortality pattern and associated risk factors: a case-control study at the Princess Marie Louise Children’s Hospital in Accra, Ghana

**DOI:** 10.1186/s12887-016-0682-y

**Published:** 2016-08-31

**Authors:** Edem M. A Tette, Mame Y. Nyarko, Edmund T. Nartey, Margaret L. Neizer, Adolph Egbefome, Fredua Akosa, Richard B. Biritwum

**Affiliations:** 1 Department of Community Health, School of Public Health (Korle Bu Campus), University of Ghana, P. O. Box 4236, Accra, Ghana; 2 Princess Marie Louise Children’s Hospital, P. O. Box GP 122, Accra, Ghana; 3 Centre for Tropical Clinical Pharmacology and Therapeutics, School of Medicine and Dentistry, University of Ghana, P. O. Box 4236, Accra, Ghana; 4 Legon Hospital, University of Ghana, P. O. Box 25, Legon, Accra Ghana

**Keywords:** Under 5 mortality, Malnutrition, Child deaths, Diarrhoea, ETAT, Pneumonia

## Abstract

**Background:**

Millions of children under the age of 5 years die every year. Some of these deaths occur in hospitals and are related to both clinical characteristics and modifiable risk factors. This study investigates the association between malnutrition and mortality and profiles the presenting features in a case-control study of children under 5 years of age who attended Princess Marie Louise Children’s Hospital (PML) in 2011.

**Methods:**

A total of 120 cases of children under the age of 5 years who were admitted to hospital and died there were matched by sex and age to 120 controls who were children who survived on 1:1 basis from a record of patients admitted to PML in 2011. Data on socio-demographic and clinical characteristics were extracted from the medical records of the study participants. The association between malnutrition and mortality was determined by conditional logistic regression reported as odds ratios (OR) and their 95 % confidence interval (95 % CI). *P* < 0.05 was considered significant in all analyses.

**Results:**

Malnutrition was significantly associated with mortality in children under-5 years of age attending PML. In the adjusted analysis, the odds of dying was significantly higher in malnourished children compared with well-nourished children (adjusted OR = 4.32 [95 % CI, 1.33–13.92], *p* = 0.014]). In addition, a previous episode of diarrhoea within the last year was associated with mortality (adjusted OR = 7.25 [95 % CI, 1.68–31.22], *p* = 0.008). The proportion of patients with noisy or difficulty breathing, pallor, lethargic appearance, ill-looking appearance, febrile convulsion, altered sensorium, skin lesions, hepatomegaly or oedema was significantly higher among cases than in controls (*p* < 0.05).

**Conclusions:**

Malnutrition and a previous episode of diarrhoea within the last year were the main risk factors for mortality. Efforts to prevent malnutrition and diarrhoea must be intensified and a protocol to follow-up diarrhoea patients may be beneficial. Six out of the nine clinical features that were proportionally higher in children who died than those who survived, are captured by the Emergency Triage Assessment and Treatment (ETAT) screening protocol as emergency or priority signs, giving credence to the use of ETAT in this setting. Thus education of health professionals on the use of the tool to triage patients should be on-going. However, further studies are needed to establish whether the other clinical signs are consistently associated with mortality and if so, whether they can be included among triage criteria, danger signs or in a prognostic scoring system for this setting.

## Background

There has been a significant reduction in child mortality world-wide since the Millennium Development Goals (MDGs) were first launched [1]. Globally, under-five mortality declined by 53 % from 91 per 1000 live births to 43 by 2015, however, it remains high in sub-Saharan Africa and many countries in the region were unable to reach the reduction stipulated by MDG 4 targets [2]. In Ghana, under-5 mortality has declined by 61 % from 155 deaths per 1000 live births in the 1980s to 60 deaths per 1000 live births by 2014 but it fell short of the the targeted two thirds reduction to 40 deaths per 1000 live births by the year 2015 [3].

Although it has been argued that facility based data do not capture all the deaths occurring in a community they provide a means to study modifiable risk factors for preventing child mortality [4, 5]. Modifiable risk factors refer to events, actions or omissions contributing to the death of a child or to substandard care of a child who died, and which by means of locally achievable interventions, can be modified [5, 6]. They are categorized into caregivers or family related problems, administrative problems and medical personnel-related problems [5–8]. In countries where mortality audits and child death reviews are carried out regularly, and steps have been taken to address these modifiable factors, they have been an invaluable source of information used to prevent child deaths and improve care [5, 9]. This is why it is important to identify factors associated with mortality in hospitalised children periodically.

Every year, many children in middle and low-income countries are admitted into hospital since it is expected that 10–20 % of sick children presenting to primary care facilities in developing countries, constituting the most severely ill, may require referral for hospital care in a district hospital [10]. However, paediatric mortality in hospitals in the developing world remains unacceptably high even in spite of good care seeking behaviours [11–13]. This has generated discussion about improving systems of care for hospitalized children, particularly in Africa [10, 12, 13]. Prioritizing the recognition and treatment of emergent illness in infants and children through Emergency Triage, Assessment and Treatment (ETAT) and other similar system based improvements, has demonstrated success in decreasing hospital mortality.

Emergency Triage, Assessment and Treatment is a process used to quickly evaluate patients and sort them into groups based on the urgency with which they need medical attention [14–16]. It uses presenting clinical features to categorise the patients as emergency, priority and non-urgent [14, 16, 17]. The clinical criteria used include signs of respiratory distress, shock, severe malnutrition and dehydration among others. A study in Malawi showed a drop in mortality, from 10–18 % to 6–8 % after ETAT was introduced to one of their teaching hospitals [18]. Similarly, another study from Malawi, showed a reduction in early mortality from 47.6 to 37.9 deaths per 1000 admissions in an Under-5 Clinic after implementing the triage system [16]. These reductions in mortality were also attributed to involvement of paediatricians or more experienced clinical staff as well as functional and structural changes to the emergency service, which were implemented concurrently with the triage system [16, 18].

Further information to identify risk factors associated with the unacceptably high hospital mortality is needed. Malnutrition is one of the factors that have consistently been associated with paediatric mortality in hospitals in Africa and elsewhere [19–22]. A recent study in Malawi found that moderate and severe underweight was associated with mortality from pneumonia [19]. A study in Kilifi, Kenya also found that severe underweight was a risk factor for post-discharge mortality [21]. The risk of dying from diarrhoeal diseases has also been associated with malnutrition [22]. Thus, malnutrition is one of the identifiable risk factors for paediatric hospital mortality that should be considered when assessing the risk of dying upon admission.

Unfortunately, published data on the association between malnutrition and other factors associated with paediatric mortality in hospitals in Ghana is limited. Available information is mostly from mortality studies carried out at the main teaching hospitals and though they describe the pattern of child mortality, they lack detail on risk factors associated with mortality and current data [23, 24]. Furthermore, although mortality data is available in the annual reports of most hospitals they have not been critically examined and put in context with risk factors.

In this study, the primary objective was to determine the association between malnutrition and mortality in children under-5 years who were admitted to PML between 1^st^ January 2011 and 31^st  ^December 2011 using a case-control study design. In addition, we profiled the clinical features and reported socio-demographic characteristics of the patients and the diagnosis on admission of children who died and those who survived.

## Methods

### Setting

This study was conducted at the Princess Marie Louise Children’s Hospital (PML) in Accra. PML is a 74-bed government hospital located in the commercial part of the capital, Accra. It is the second largest provider of specialist paediatric services in the capital and provides both primary and secondary care. It serves an inner city population along the coast of the southern border of the country and the rest of Accra, providing both primary care and specialised services in paediatrics. Thus, parents do not need a referral to bring their children to the hospital. The hospital is also regarded as the hospital for malnourished children because it has the largest facility for rehabilitating malnourished children in the country.

### Study population

Cases and controls were obtained. Cases were children under the age of 5 years who died during hospitalization and controls were the next child listed in the admission book who survived. Controls were age and sex matched to each individual case. If the control’s medical notes were missing, the next listed age and sex matched survivor with available medical notes was recruited as a control. The ages of the controls were matched within ± 1 month of the actual age of the cases. Children aged 5 years or more were excluded from the cases and children with missing medical notes were excluded from the study.

### Data collection methods and instruments

A data collection instrument containing socio-demographic and clinical parameters was used to collect data from the clinical folders (case notes) of study participants. Nutritional status was determined. Severe Acute Malnutrition (SAM) is defined as children with an admission weight-for-height <70 % of the National Centre for Health Statistics (NCHS) median value or below - 3 Z-score with or without bilateral pitting oedema and/or children aged 6 months and above with Mid Upper Arm Circumference (MUAC) less than 11.5 cm [25, 26].

Moderate Acute Malnutrition (MAM) is defined as children with a weight-for-length (or height) < 80 % but 70 % of the NCHS median value or between -2 and -3 Z-scores and/or children aged 6 months and above with MUAC of 11.5 cm to <12.5 cm [25, 26]. Though weight-for-height measurements are preferred for assessing malnutrition, for the purposes of this study we assessed malnutrition using weight-for-age measurements since height measurements are not routinely available. Thus malnutrition refers to a weight-for-age of < -2 Z-score as computed by WHO anthro-calculator [27]. We did not differentiate between severe and moderate underweight.

The presenting features of disease on admission including those clinical features in the ETAT triage criteria were obtained. In addition, the cause of illness or final diagnosis of patients who died and patients who survived were obtained from the case notes and have been recorded as diagnosis on admission. The final diagnosis of both the cases and controls were reviewed by the principal investigator and reduced to a single diagnosis, although it is well known that children from developing countries may have two or more diseases at the same time. Discrepant cases were discussed with team members and a consensus was reached. The International Classification of Diseases (ICD) 10 criteria provides a standard way of classifying the causes of death, however, limitations associated with the use of this criteria in developing countries have been described [5, 28]. Thus the causes of mortality were classified using modified forms of ICD 10 criteria and WHO criteria as others have done [5, 7, 24, 28].

### Data handling and statistical analysis

The data were entered, cleaned and managed using Microsoft Access (Microsoft Corporation, Edmond, Washington) and analyzed using Stata SE 11 (Stata Corporation, College Station, Texas). Continuous variables were reported as mean ± SD or median with interquartile range if not normally distributed. Categorical variables were reported as percentages, and the test statistic was based on a McNemar test. Odds ratios were calculated with conditional logistic regression before and after adjustment for potential confounding factors. A univariate analysis was done to determine the association between mortality and socio-demographic, clinical and modifiable factors. Among the factors studied were nutritional status, National Health Insurance Scheme (NHIS) registration status, referral source, previous hospital admission within the last year, previous hospital visits within the last year and previous episode of diarrhoea within the last year. Others were, reported feeding problems, duration of symptoms before presentation to hospital, haemoglobin level, white blood cell count (WBC), neutrophil count, lymphocyte count, platelet count and temperature at presentation. We then generated a multivariate model to determine the association between malnutrition and mortality including only potential confounders from the above list with *p*-value <0.20 in the univariate analysis. The subsequent multivariate model was adjusted on the selected confounders (referral source, previous hospital admission within the last year, previous episode of diarrhoea within the last year, reported feeding problems, duration of symptoms before presentation to hospital and nutritional status. All statistical tests were 2-sided, and *p* < 0.05 was considered statistically significant. As part of a larger of a larger Child Mortality Study, this study examined clinical characteristics in more detail and risk factors for mortality over a 1 year period using case-control methodology following discussion with a biostatistician because comprehensive data was only extracted from and thus available in this group of children.

## Results

### Demographic characteristics of study participants

Out of a total of 4677 admissions between 1st January 2011 and 31st December 2011, 130 deaths occurred in children under 5 years of age. Ten (10) case notes of the children under 5 years of age who died were missing and were not included in the study. Altogether 240 medical records were retrieved and analysed comprising 120 children under 5 years of age who died on admission (cases) and 120 age and sex matched children under 5 years of age who were discharged from the hospital (controls). Table 1 shows the demographic characteristics of the study participants. The majority of the children (80.0 %) were below 2 years and most of them were not registered with the NHIS (70.6 % cases and 63.2 % controls) (Table 1).

**Table 1:** Socio-demographic characteristics of cases and controls admitted in PML between 1st January 2011 and 31st December 2011

Characteristic	Cases number, %	Controls number, %
Age of child, months	*N* = 120	*N* = 120
< 1	9 (7.5)	9 (7.5)
1–5	36 (30.0)	36 (30.0)
6–11	30 (25.0)	30 (25.0)
12–23	21 (17.5)	21 (17.5)
24–59	24 (20.0)	24 (20.0)
Gender	*N* = 120	*N* = 120
Male	60 (50.0)	60 (50.0)
Female	60 (50.0)	60 (50.0)
NHIS registration	*N* = 102	*N* = 117
Registered	30 (29.4)	43 (36.8)
Not registered	72 (70.6)	74 (63.2)

*N* = Total number of responses

### Presenting features and clinical characteristics of study participants

Table 2 provides a summary of the presenting features of disease among the cases and controls. Fever was the most frequent presenting feature in both cases and controls (83.3 % and 86.7 % respectively). The proportion of patients with noisy or difficulty in breathing, pallor, lethargic appearance, ill-looking appearance, febrile convulsion, altered sensorium, skin lesions, hepatomegaly or oedema was significantly higher in cases than in controls (*p* < 0.05) (Table 2).

**Table 2:** Presenting features of disease in cases and controls after matching

Presenting features of disease	Cases number, %	Controls number, %	*p*-value
Fever	100 (83.3)	104 (86.7)	0.450
Vomiting	61 (50.8)	48 (40.0)	0.069
Cough	54 (45.0)	49 (40.8)	0.466
Diarrhoea	58 (48.3)	45 (37.5)	0.053
Noisy/Difficulty in breathing	52 (43.3)	25 (20.8)	<0.001
Pallor	67 (55.8)	10 (8.3)	<0.001
Poor feeding	34 (28.3)	29 (24.2)	0.465
Appearance (Lethargic)	36 (30.0)	10 (8.3)	<0.001
Appearance (Ill-looking)	31 (25.8)	7 (5.8)	<0.001
Febrile convulsion	22 (18.3)	7 (5.8)	0.004
Altered sensorium/unconscious	22 (18.3)	3 (2.5)	<0.001
Skin Lesions	17 (14.2)	6 (5.0)	0.008
Jaundice	7 (5.8)	7 (5.8)	0.763
Hepatomegaly	10 (8.3)	1 (0.8)	0.007
Oedema	10 (8.3)	0	0.002
General Malaise	4 (3.3)	3 (2.5)	0.706
“Coca-Cola” urine (dark urine)	3 (2.5)	3 (2.5)	1.000
Developmental delay	3 (2.5)	1 (0.8)	0.317
Bleeding	2 (1.7)	0	0.500
Hypoglycaemia	2 (1.7)	0	0.500
Splenomegaly	2 (1.7)	0	0.500
Abdominal Pain	1 (0.8)	0	1.000
Headache	1 (0.8)	0	1.000

Table 3 shows the diagnosis on admission in the cases and controls. Pneumonia was the most common diagnosis on admission among the cases (21.7 %) while gastroenteritis was the most common diagnosis on admission among the controls occurring in 22.5 % (Table 3). Altogether, seven diseases, namely pneumonia, gastroenteritis, malaria, septicaemia, malnutrition, HIV infection and meningitis accounted for 84.2 % of the diagnosis on admission in cases and 76.7 % of the diagnosis on admission in controls.

**Table 3:** Primary cause of death in cases and primary cause of illness in controls. Admissions were in PML hospital, Accra between 1st January 2011 and 31st December 2011

Disease diagnosis	Primary cause of illness (*N* = 120) number, %	Primary cause of illness Controls (*N* = 120) number, %
Pneumonia	26 (21.7)	14 (11.7)
Gastroenteritis	15 (12.5)	27 (22.5)
Malaria	15 (12.5)	21 (17.5)
Septicaemia	15 (12.5)	21 (17.5)
Malnutrition	14 (11.7)	12 (10.0)
HIV Infection	10 (8.3)	-
Meningitis	6 (5.0)	1 (0.8)
Undiagnosed	4 (3.3)	-
Anaemia	3 (2.5)	1 (0.8)
Other acute respiratory illness	2 (1.7)	7 (5.8)
Liver disease	2 (1.7)	-
Congenital heart disease	2 (1.7)	-
Down’s syndrome	1 (0.8)	1 (0.8)
Chronic liver disease	1 (0.8)	-
Haemorrhagic disease of newborn	1 (0.8)	-
Tuberculosis	1 (0.8)	-
Nephrotic syndrome	1 (0.8)	-
Asthma	1 (0.8)	3 (2.5)
Sickle cell disease	1 (0.8)	2 (1.7)
Seizure/convulsion	-	3 (2.5)
Swallowed blood	-	1 (0.8)
Measles	-	1 (0.8)
Fracture of the left femur	-	1 (0.8)
Urinary tract infection	-	1 (0.8)
Sudden collapse	-	1 (0.8)
Poisoning	-	1 (0.8)
Impetigo with right breast abscess	-	1 (0.8)

Table 4 shows some clinical characteristics of the cases and controls. Anaemia was present in 69 (77.5 %) cases and 78 (72.9 %) controls while malnutrition was present in 75 (67.0 %) cases and 32 (27.6 %) controls (Table 4). Previous episodes of diarrhoea within the last year occurred in 40.5 % (*n* = 32) of cases compared with 15.7 % (*n* = 16) of controls (Table 4).

**Table 4:** Clinical characteristics of case and control subjects after matching

Characteristics	Case subjects number, %	Control subjects number, %
Referral source	*N* = 120	*N* = 120
Self	91 (75.8)	110 (91.7)
Government /Private hospital or polyclinic	29 (24.2)	8 (8.3)
Previous hospital visits within the last year	*N* = 120	*N* = 120
Yes	43 (35.8)	41 (34.2)
Previous hospital admission within the last year	*N* = 120	*N* = 120
Yes	18 (15.0)	8 (6.6)
Reported feeding problems	*N* = 120	*N* = 120
Yes	15 (12.5)	22 (18.3)
Previous episodes of diarrhoea within the last year	*N* = 79	*N* = 102
Yes	32 (40.5)	16 (15.7)
Duration of symptoms before presentation, days	*N* = 109	*N* = 117
1–2 days	28 (25.7)	56 (47.9)
2 days	81 (74.3)	61 (52.1)
Nutritional status (Weight -for-age)	*N* = 112	*N* = 116
Well nourished (z-score SD ≥ -2)	37 (33.0)	84 (72.4)
Malnourished (z-score SD < -2)	75 (67.0)	32 (27.6)
Temperature measurement at presentation	*N* = 115	*N* = 120
Normal (<37.5oC)	32 (27.8)	41 (34.2)
Fever (≥37.5 °C)	83 (72.2)	79 (65.8)
Haemoglobin level at presentation	*N* = 89	*N* = 107
Normal haemoglobin level (≥11.0 g/dL)	20 (22.5)	29 (27.1)
Anaemic (<11.0 g/dL)	69 (77.5)	78 (72.9)
WBC count at presentation	*N* = 84	*N* = 105
Normal (2.5 x 10^3^-8.5 x 10^3^/μ L)	18 (21.4)	32 (30.5)
High (>8.5 x 10^3^/μ L)	66 (78.6)	73 (69.5)
Neutrophil count at presentation	*N* = 78	*N* = 96
Normal (≤75.0 %)	68 (87.2)	89 (92.7)
High (>75.0 %)	10 (12.8)	7 (7.3)
Platelet count at presentation	*N* = 71	*N* = 86
Normal (≤400 x 10^3^/μ L)	39 (54.9)	47 (54.7)
High (>400 x 10^3^/μ L)	32 (45.1)	39 (45.3)
Lymphocyte count at presentation	*N* = 78	*N* = 96
Normal (≤60.0 %)	68 (87.2)	79 (82.3)
High (>60.0 %)	10 (12.8)	17 (17.7)

### Factors associated with mortality in children under 5 years of age

Table 5 also shows the socio-demographic and clinical factors associated with mortality in children under 5 years of age. In the adjusted conditional regression model, malnutrition and a previous episode of diarrhoea within the last year were significantly associated with mortality. The odds of dying was higher in children under 5 years of age who were malnourished compared with well-nourished children (adjusted OR 4.44 [95 % CI, 1.31–15.06], *p* = 0.017) (Table 5). Similarly, the odds of dying was higher in children under 5 years of age with a previous episode of diarrhoea within the last one (1) year compared with children with no previous episode of diarrhoea within the last one (1) year (adjusted OR, 6.93 [1.50–32.08], *p* = 0.013) (Table 5).

**Table 5:** Socio-demographic and clinical factors associated with child mortality in a matched case control study at PML hospital in Accra, Ghana

Characteristic	Crude OR [95 % CI]	*p*-value	Adjusted OR [95 % CI]^1^	*p*-value
Referral source				
Hospital/clinic referral	3.38 [1.53–7.43]	0.003	1.43 [0.40–5.19]	0.584
Self-referral	1.00		1.00	
Previous hospital visit within the last year				
Yes	1.08 [0.62–1.89]	0.777	-	-
No	1.00			
Previous hospital admission within the last year				
Yes	2.67 [1.04–6.81]	0.040	3.08 [0.48–19.88]	0.238
No	1.00		1.00	
Previous episode of diarrhoea within the last year				
Yes	5.50 [1.90–15.96]	0.002	6.93 [1.50–32.08]	0.013
No	1.00		1.00	
Reported feeding problem				
Yes	0.61 [0.29–1.29]	0.198	0.88 [0.21–3.70]	0.862
No	1.00		1.00	
Duration of symptoms before presentation				
1–2 days	2.5 [1.35–4.65]	0.004	2.00 [0.56–7.11]	0.286
> 2 days	1.00		1.00	
Nutritional status (WAZ)				
Malnourished (z-score SD < -2)	4.33 [2.31–8.12]	<0.001	4.44 [1.31–15.06]	0.017
Well nourished (z-score SD ≥ -2)	1.00		1.00	
NHIS registration				
Not registered	1.38 [0.79–2.42]	0.260	-	-
Registered	1.00			
Haemoglobin level category				
Anaemic (<11.0 g/dL)	1.25 [0.59–2.67]	0.565	-	-
Normal haemoglobin level (≥11.0 g/dL)	1.00			
Temperature measurement at presentation				
Fever (≥37.5oC)	1.39 [0.76–2.55]	0.288	-	-
Normal (<37.5oC)	1.00			
WBC count at presentation				
High (>8.5 x 10^3^/μ L)	1.78 [0.79–4.02]	0.267		
Normal (2.5 x 10^3^-8.5 x 10^3^/μ L)	1.00			
Neutrophil count at presentation				
High (>75.0 %)	1.50 [0.42–5.32]	0.530	-	-
Normal (≤75.0 %)	1.00			
Platelet count at presentation				
High (>400 x 10^3^/μ L)	1.22 [0.51–2.95]	0.655	-	-
Normal (≤400 x 10^3^/μ L)	1.00			
Lymphocyte count at presentation				
High (>60.0 %)	1.00 [0.32–3.10]	1.000		
Normal (≤60.0 %)	1.00			

^1^Variables with *p* < 0.2 in the univariate analysis were included in the multivariate analysis

*WAZ* weight-for-age z- score, *OR* odds ratio, *CI* confidence interval

## Discussion

Malnutrition determined by a weight for age of < -2 z-score was the major clinical characteristic associated with mortality in children under 5 years of age in this study. Similar findings have been consistently reported in other studies on mortality [7, 8, 24, 28]. Fortunately, an analysis of the changing patterns of disease and mortality at PML by Tette et al. also indicates a reduction in mortality due to malnutrition between 2003 and 2013 [29]. This makes it imperative that cases of malnutrition among those who attend the hospital for other reasons are picked up early by doing height and Mid Upper Arm Circumference (MUAC) measurements at the nurses’ desk to reduce missed opportunities for intervention that often characterise health facilities in the developing world [30, 31]. Furthermore, efforts should be made to prevent malnutrition using the new Global Nutrition Targets 2025 [32].

Though our study did not find an association between anaemia and mortality, we did find anaemia present in over 70 % of cases and controls. Thus interventions to reduce malnutrition should also include specific policies for reducing anaemia in children under 5 years of age since both conditions have an effect on cognitive development, human capital and transmission of intergenerational poverty in the long term [33].

This study also showed that a previous episode of diarrhoea within the last year was associated with mortality in the multivariate analysis. A similar report was made by researchers in Kenya [34]. The quality of care received by patients with diarrhoea also needs to be further examined to ensure that the presence of underlying conditions such as HIV infection, malnutrition and mal-absorption, which can cause recurrent diarrhoea are identified, and treated [35, 36]. In addition, a policy guideline for following up patients with diarrhoea to reduce mortality is needed. At the same time, promotion of cost effective interventions in primary care should be on-going emphasising home use of oral re-hydration salt (ORS), early presentation to hospital, zinc supplementation, optimizing breastfeeding and improving personal and community hygiene and sanitation [37].

Although the present results indicate no association between referral source and mortality in the multivariate analysis, it is likely that the open access policy at PML, which allows parents to walk in without a referral, offers a safety net for parents and should be encouraged. Similarly, this study found no association between duration of symptoms before presentation and mortality at the multivariate level, although it was significant at the univariate level and it has been reported that children who present late to hospital have poorer outcome [11, 12, 36].

Emergency Triage Assessment and Treatment trains health professionals to recognise emergency signs and also priority clinical signs so that children who are in need of urgent attention can be prioritised in order to improve the quality of care children receive in hospitals [18]. The emergency signs included in the triage criteria are severe respiratory distress, central cyanosis or obstructed breathing, shock including cold hands, delayed capillary refill time, a weak and fast pulse, comma, convulsion and severe dehydration i.e., lethargy, sunken eyes and very slow skin pinch [14]. On other hand, the priority signs are visible and include tiny infant, temperature, severe trauma, severe pallor, poisoning, severe pain, respiratory distress, restless, continuously irritable or lethargic, urgent referral, severe wasting (severe malnutrition), oedema of both feet, and major burns. If no emergency or priority signs are found, the child must wait in line to see a clinician and is labelled as ‘queue’ or non-urgent [14, 17]. The WHO has recently revised the ETAT protocol and introduced new guidance regarding fluid management and the assessment and management of shock [38].

In this study the presenting features that were significantly higher in cases than in controls were noisy or difficulty in breathing, pallor, lethargy, ill- looking appearance, febrile convulsion, altered sensorium, skin lesions, hepatomegaly, and oedema. Out of these nine (9) presenting features, six (6) are captured by the ETAT screening protocol as either emergency or priority signs [39]. This therefore gives further credence to the use of ETAT in our current settings and therefore re-enforces calls for efforts to disseminate the training to other hospitals. However, further studies are needed to establish whether ill-looking appearance, hepatomegaly and skin lesion as presenting features are consistently associated with mortality, and to find out the diseases they represent to determine whether they need to be included in the triage criteria, or as danger signs to alert parents in this setting. These signs can also be considered when developing a prognostic scoring system for this setting as developing such a system has been the focus of current research to improve the quality of care for sick children [40, 41].

There were some instances of missing information and inadequate documentation, making it difficult to obtain or comment on some of the data. Some patients had more than one diagnosed condition; however, for the purposes of this study a single diagnosis was given and we may have lost some important information. Thus, the data presented on the cases here is methodologically different from the data we presented elsewhere [29] from the Child Mortality Study and it includes all cases with available medical records. Upgrading the current mortality meeting through the use of Medical Audit forms such as the U5PIP or standardized clerking sheets can provide more detailed information [7, 10]. This, together with the computerisation of the records department will enable administrative and other modifiable factors contributing to child mortality not only to be more readily identified, but also addressed and monitored.

Since the hospital has a specialised unit for malnourished children, the cases of malnutrition may have been over-represented. Although weight-for-height and Mid Upper Arm Circumference (MUAC) are the recommended measurements for assessing moderate and severe acute malnutrition, these measurements were unavailable for most patients because they are not routinely done in all patients therefore we used weight-for-age criteria.

## Conclusions

Malnutrition and a previous episode of diarrhoea were the main risk factors for mortality. Efforts to prevent these diseases must be intensified. Education on danger signs and early presentation must be strengthened at the hospital and in communities that use the facility. Most of the presenting features that were proportionally higher in cases than in controls were captured by the ETAT screening protocol. However, further studies are needed to determine other clinical signs of conditions that are consistently associated with mortality in this setting so they can be included as part of the triage criteria, danger signs or in a prognostic scoring system for this setting.

## Abbreviations

ETAT: Emergency Triage Assessment and Treatment
ICD: International Classification of Diseases
MAM: Moderate Acute Malnutrition
MDGs: Millennium Development Goals
MUAC: Mid-Upper Arm Circumference
NCHS: National Centre for Health Statistics
NHIS: National Health Insurance Scheme
ORS: Oral re-hydration salt
PML: Princess Marie Louise Children’s Hospital
SAM: Severe Acute Malnutrition
WHO: World Health Organisation
